# Cerebrospinal Fluid Calcium Balance in Tick-Borne Encephalitis: A Preliminary Study and Future Research Directions

**DOI:** 10.3390/biomedicines13020337

**Published:** 2025-02-02

**Authors:** Gabriela Trojan, Anna Moniuszko-Malinowska, Karolina Orywal, Ewelina Kruszewska, Barbara Mroczko, Anna Grzeszczuk, Piotr Czupryna

**Affiliations:** 1 Department of Infectious Diseases and Neuroinfections, Medical University of Bialystok, 15-089 Białystok, Poland; annamoniuszko@op.pl (A.M.-M.); kruszewska.ewelina@gmail.com (E.K.); anna.grzeszczuk@umb.edu.pl (A.G.); avalon-5@wp.pl (P.C.); 2 Department of Biochemical Diagnostics, Medical University of Bialystok, 15-089 Białystok, Poland; orywalk@umb.edu.pl (K.O.); barbara.mroczko@umb.edu.pl (B.M.)

**Keywords:** calcium, ion-specific assays, diagnostic marker, neuroinflammation, neurodegenerative processes, tick-borne encephalitis, tick-borne infections, cerebrospinal fluid, biomarker, disease severity

## Abstract

**Introduction**: Calcium homeostasis is essential for neurophysiological functions, with dysregulation implicated in neurodegenerative diseases. Recent studies suggest that specific viral brain infections, such as tick-borne encephalitis, can initiate neuronal loss and subsequent neurodegenerative changes. This study examines alterations in calcium levels within the cerebrospinal fluid (CSF) of patients with tick-borne encephalitis (TBE). **Objectives**: To evaluate the concentration of calcium in the CSF of TBE patients and assess its potential as a diagnostic marker for disease severity. **Materials and Methods**: CSF samples were collected from 42 subjects (11 controls, 20 with TBE, 11 with other forms of meningitis). Calcium levels were measured using the Alinity c analyzer. Statistical analyses included the Shapiro–Wilk test, Mann–Whitney U test, and ROC curve analysis. **Results**: Calcium levels were significantly lower in TBE patients compared to controls (mean 0.85 mmol/L vs. 0.98 mmol/L). Lower calcium levels were associated with milder cases of TBE. ROC analysis (AUC 0.802, *p*-value 0.0053) supports the diagnostic utility of calcium concentration in differentiating TBE severity. The optimal cut-off value for calcium was >3.09 mg/dL, with a sensitivity of 84.62% and specificity of 71.43%. These findings further emphasize the potential of calcium as a diagnostic marker for TBEV. **Conclusions**: The observed differences in CSF calcium levels between mild and severe TBE cases highlight its potential as a diagnostic marker. Further research is warranted to elucidate calcium’s role in TBE, aiming to improve clinical management and reduce complications. We emphasize that this study is one of the first to propose calcium levels as a potential biomarker for assessing the severity of tick-borne encephalitis, offering a new perspective in the diagnostic approach to this infection.

## 1. Introduction

Calcium (Ca) is a vital element for the proper functioning of the human body, especially in the nervous, muscular, and skeletal systems. It accounts for about 2% of the total body mass, with 99% of this calcium present in bones as hydroxyapatite crystals, which make up the mineral component of bones [1,2]. The remaining 1% is found in soft tissues and extracellular fluid (ECF), including blood [1,2]. Under normal physiological conditions, blood calcium levels are influenced by: dietary calcium intake, calcium absorption from the digestive tract into the bloodstream, calcium excretion via urine and calcium deposition or release from bones [2]. Calcium imaging in dendritic components is essential for directly examining age- or disease-related changes that occur during synaptic activation [3]. The normal range for total calcium concentration in the body remains within 8.5–10.5 mg/dL (2.12–2.61 mmol/L) [1]. The calcium concentration in the cerebrospinal fluid (CSF) of adults is consistently around 5.0 mg per 100 cc of fluid (what is exactly 5 mg/dL and 1247 mmol/L). This amount is approximately half of the calcium content found in the blood plasma or serum of similar individuals [4]. Other scientific sources report the following expected ranges for calcium concentration in cerebrospinal fluid: Kandel cites 2.1 mEq/L (which is equivalent 4,2 mg/dL and 1.047 mmol/L) [5], while Romaris provides values around 4.95 mg/dL (1.234 mmol/L) [6]. The sources agree that the level of calcium in CSF should be relatively stable, thus any deviations from this norm should be considered indicative of calcium homeostasis disorders [4,5,6,7]. In conclusion, the range cited by the literature as normal for a healthy individual is between 4.2 mg/dL and 5 mg/dL (1.047 mmol/L and 1.247 mmol/L).

The imbalance of intracellular calcium levels is a crucial element in neurodegeneration. This dysregulation causes the improper activation of calcium-dependent mechanisms, eventually leading to cell death [8,9,10,11].

Elevated calcium levels in CSF have been observed in subarachnoid hemorrhage [12], ischemic insults, injury, neurodegenerative pathologies [13], epilepsy [14] psychiatric disorders [4]. Lowered calcium levels have been documented, i.e., in sepsis [15]. Reduced calcium levels can be observed in Fahr’s syndrome, presenting clinically with persecutory delusions, limb tremors, and dysarthria, as well as in cases of postpartum psychosis [4].

The main site of CSF production is the choroid plexuses in the brain’s lateral ventricles, with a smaller amount also generated by the tissue lining the brain’s ventricles and the subarachnoid space. The CSF flows from the lateral ventricles through the foramen of Monroe to the third ventricle, then through the aqueduct of Sylvius to the fourth ventricle, and finally through the foramen of Magendie and the two lateral foramina of Luschka into the subarachnoid space [16].

The initial stage of CSF production involves plasma filtration from the blood vessels within the choroid plexus, driven by an osmotic pressure gradient. This is followed by the active transport of ions via transport proteins located in the membranes of the choroid plexus epithelial cells [16].

Epithelial cells in the choroid plexus contribute to the blood–cerebrospinal fluid barrier (BCSFB). These cells are connected by tight junctions, which prevent molecules from freely passing into the forming CSF. Additionally, these epithelial cells possess various transport systems that facilitate the movement of ions and nutrients into the CSF and the removal of harmful substances from the CSF originating from the nervous system [16].

The tick-borne encephalitis virus (TBEV), a member of the Flavivirus genus within the *Flaviviridae* family, is endemic to a broad region extending from central and northern Europe to Siberia and Japan. This virus is naturally transmitted among tick vectors, predominantly those within the *Ixodes persulcatus* complex, and various vertebrate hosts. Over the last thirty years, TBEV has become a prominent tick-borne flavivirus in Europe and Asia, representing an increasing public health concern with an estimated annual incidence of approximately 13,000 human cases [17,18,19]. In 2019, there were 3411 reported cases in Europe, with a case fatality rate of 0.7% [20]. Tick-borne encephalitis (TBE) remains a major cause of viral encephalitis in Europe and Asia, causes clinical illness in over 20,000 individuals [17,21]. The rising prevalence and transmission of tick-borne diseases present significant public health [17].

A growing trend in tick-borne encephalitis (TBE) cases can also be observed in Poland. According to official data from the National Institute of Public Health, the confirmed number of cases in 2022 was 446, while in 2023 this number increased to 663. The highest number of cases was recorded in the Podlaskie Voivodeship, with 213 cases (accounting for 32.13% of the total), followed by the Masovian Voivodeship with 134 cases (20.21%). The Warmian–Masurian Voivodeship ranked third with 86 cases (12.97% of the total). Both the Podlaskie and Warmian–Masurian Voivodeships are considered endemic areas for the tick-borne encephalitis virus [22,23]. However, it is believed that these figures are still underestimated, and the actual incidence of TBE is significantly higher.

Our understanding of the mechanisms by which the tick-borne encephalitis virus (TBEV) induces neurological damage remains incomplete. The virus initially replicates in dendritic cells located at the site of the tick bite. These infected dendritic cells then migrate to adjacent lymph nodes, facilitating the entry of the virus into the bloodstream and possibly enabling it to cross the blood–brain barrier, resulting in brain lesions. The subsequent neurological damage is presumed to be a consequence of the virus infecting neurons and various other cellular components within the central nervous system (CNS). This includes resident glial cells, such as microglia and astrocytes, as well as infiltrating immune cells like CD8+ T cells and natural killer cells, which play pivotal roles in the innate immune response [21].

Numerous studies have been undertaken to address the gaps in understanding regarding TBEV-induced damage in the human brain and the distinct innate immune responses of human neurons and astrocytes that influence the virus’s tropism in the brain. These efforts aim to elucidate the infection mechanisms and to develop new therapies, focusing on antiviral agents and neuroprotective strategies [21].

Alterations in calcium levels can disrupt neuronal signaling, enhance excitotoxicity, and promote inflammatory cascades, which are key components of TBE pathology. This connection highlights the rationale for focusing on calcium as a potential biomarker in our study.

## 2. Materials and Methods

Archival biological material served as the basis for this study. The medical history of patients was reviewed, and a cohort of 42 cerebrospinal fluid samples collected between 2021 and 2024 was selected. According to documentation, the cohort was divided into three groups:

(1) Group I: TBE. Each member of the group had experienced active tick-borne encephalitis virus infection (20 samples);
(2) Group II: Other etiology. Each member of the group had experienced meningitis of another etiology (11 samples);
(3) Group III: the control group (CG) (11 samples).

None of the patients had been vaccinated against TBE. Inclusion criteria for the control group involved lumbar puncture with non-inflammatory fluid parameters and a medical history devoid of indications of proliferative or neurodegenerative diseases, or trauma-related conditions.

Patients in the TBE group underwent evaluation for specific IgG and IgM antibody levels in the cerebrospinal fluid and serum. The diagnosis of the disease was established based on clinical presentation, the presence of inflammatory markers in CSF, and specific antibodies in both serum and CSF, according to the case definition. This definition includes clinical signs of meningitis, meningoencephalitis, or meningoencephalomyelitis, an epidemiological connection, CSF pleocytosis (>5 cells/dL), and evidence of recent TBEV infection indicated by specific IgM and IgG antibodies in serum [24].

Additionally, the TBE patients were categorized into two groups based on the clinical progression of the disease:

- Severe: patients with meningoencephalitis or meningoencephalomyelitis (*n* = 13: 6 women and 7 men aged between 21 and 73 years; mean 51.46 ± 17.06 years old). The diagnosis of meningoencephalitis was made based on disturbances in consciousness and/or the presence of focal neurological symptoms. The CSF examinations in this group were as follows: on admission, mean pleocytosis was 80.31 ± 97.05 cells/µL, and mean protein concentration was 84.85 ± 58.31 mg/dL.
- Mild: patients with meningitis (*n* = 7; 3 women, 4 men aged between 21 and 55 years; mean 40.43 ± 12.2 years old). The CSF examinations in this group were as follows: on admission, mean pleocytosis was 186.29 ± 133.67 cells/µL, and mean protein concentration was 57.29 ± 21.82 mg/dL.

The study was approved by the Local Bioethics Committee [APK.002.379.2024].

The third group consisted of individuals with confirmed diagnoses of bacterial or viral meningitis (based on blood and cerebrospinal fluid tests, including serology).

Total calcium levels were measured to assess the calcium concentration in the cerebrospinal fluid.

The determination of calcium concentration was conducted using the Alinity c analyzer with the Calcium Reagent Kit 07P57. The method involves the formation of calcium dye complexes that are measured spectrophotometrically. Arsenazo-III dye reacts with calcium in an acidic solution, producing a blue-purple complex. The developed color intensity is then measured at 660 nm, which is directly proportional to the calcium concentration present in the sample.

## 3. Statistical Analysis

Statistical analysis was performed using Statistica 13.3 (StatSoft) and Medcalc 11.3.

Normality of data was checked with the Shapiro–Wilk test. Groups were compared with U Mann–Whitney test and ROC curves were created. *p* < 0.05 was considered statistically significant.

## 4. Results

All obtained results fell within the measurement range of the equipment used. Calcium concentration measurements in cerebrospinal fluid were obtained in mmol/L. To convert the results to mg/dL, the following formula was used: mmol/L × 4.01 = mg/dL.

Below is the distribution of age and gender among the participants in the conducted study (Table 1).

**Figure 1:**
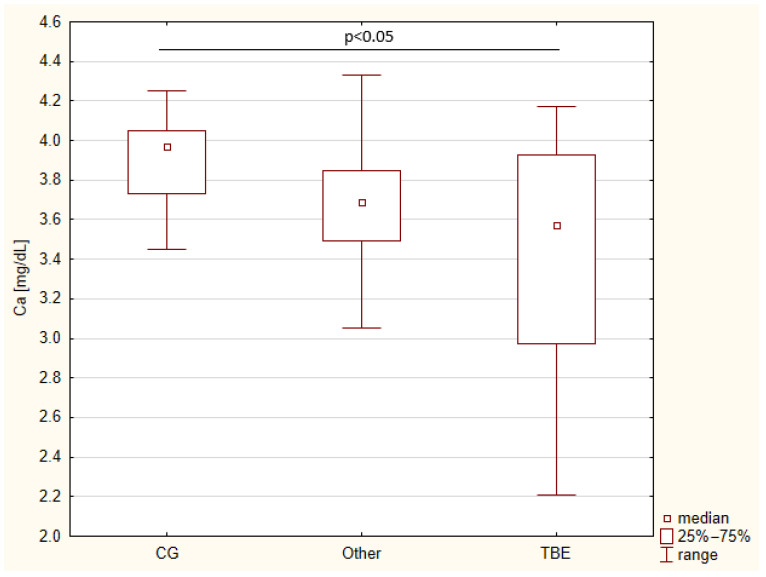
Calcium levels in CSF (box plot).

The mean calcium level in the control group was 0.975 mmol/L, while the median was 0.99 mmol/L (Table 2). Compared to the control group, the mean calcium level in individuals with tick-borne encephalitis (TBE) was significantly lower (0.98 mmol/L vs. 0.85 mmol/L) (Figure 1). The data are summarized in the table below.

A significantly lower calcium concentration is noticeable in the group with a mild course compared to the group with a severe course (Table 3). Interestingly, the averages in both subgroups are still lower than the average in the control group. The difference in calcium levels between individuals who developed severe disease and those with a mild course was significant (*p* = 0.03).

Descriptive statistics are provided for variables such as calcium concentration (both in mmol/L and mg/dL), age, sex, CRP levels, glucose, protein, albumins in CSF, cell counts, creatinine, ALT, AST, chloride levels in CSF, complications, WBC, RBC, and platelets. Calcium concentration did not correlate with other measured parameters (*p* > 0.05).

**Figure 2:**
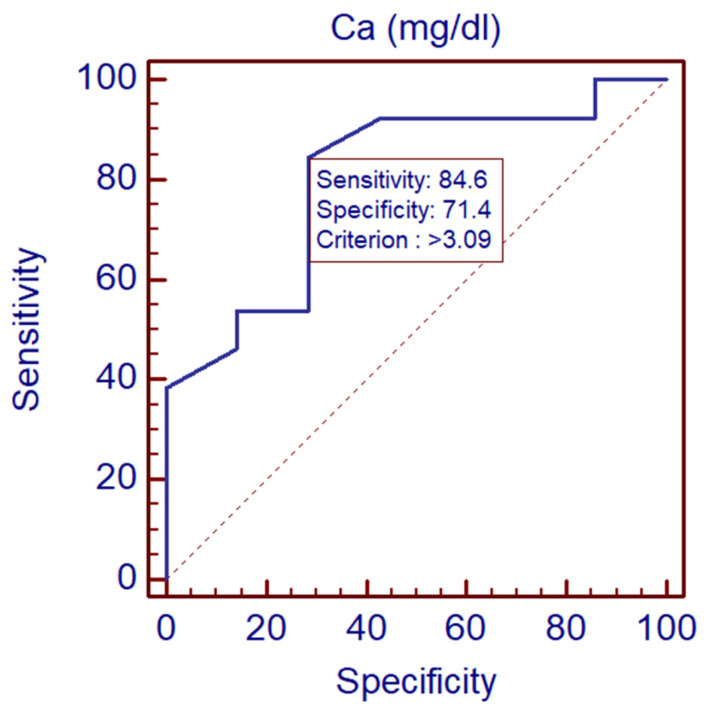
The ROC (Receiver Operating Characteristic) curve for calcium in mg/dL differentiating mild and severe TBE groups.

The ROC curve analysis for calcium in mg/dL shows an AUC (area under the curve) of 0.802, indicating a good level of test accuracy. The *p*-value was 0.0053 (Figure 2).

Specificity and sensitivity values are provided for various criterion points, with an optimal cut-off point indicated where both sensitivity and specificity are balanced. The optimal criterion value was >3.09 mg/dL with a sensitivity of 84.62% and specificity of 71.43%.

## 5. Discussion

Neuroimflammation and neurodegenerative diseases constitute a heterogeneous group of disorders characterized by the progressive deterioration of neuronal structure and function. Despite substantial advancements in comprehending their pathophysiology, a profound understanding of the underlying mechanisms and the development of novel therapeutic strategies remain imperative.

Calcium dysregulation has been implicated in a wide range of neurodegenerative diseases beyond TBE, including Alzheimer’s disease, Parkinson’s disease, and Huntington’s disease. In Alzheimer’s disease, for example, disturbances in calcium homeostasis contribute to amyloid plaque formation and neuronal death [25]. Similarly, in Parkinson’s disease, altered calcium signaling has been shown to exacerbate dopaminergic neuron degeneration, highlighting the critical role calcium plays in maintaining neuronal integrity [26]. These examples underscore the relevance of calcium dysregulation in the broader context of neurodegenerative diseases.

Tick-borne encephalitis virus (TBEV) frequently leads to meningitis, encephalitis, and meningoencephalitis. There is a critical need to investigate how the virus interacts with host factors, which will provide insights into the roles these factors play in the TBEV life cycle [17,19].

Despite advances in understanding its life cycle, details regarding host–virus interactions, receptor binding, replication mechanisms, and viral assembly remain poorly elucidated [17].

A significant proportion, ranging from 2% to 30% of cases, can progress to severe neurological complications, long-term sequelae, or even mortality. Diagnosis relies on clinical symptoms of neurological disease and the detection of virus-specific IgM and IgG antibodies. Currently, there is no targeted antiviral therapy available, and management primarily involves supportive care tailored to the diverse manifestations of the disease. Vaccination stands as an effective measure for disease prevention [19].

Recent studies have shown that tick-borne encephalitis (TBE) can be a cause of premature brain degeneration that cannot be explained by any other means [27,28,29,30,31]. Tick-borne encephalitis virus is characterized by its endemic occurrence, and the symptoms of the disease are often nonspecific and trivialized by patients and healthcare providers, particularly in the initial phase of the illness [20,28]. When the disease progresses to the second phase, patients are admitted to the hospital ward. The most common symptoms observed in patients include headaches, fevers reaching up to 40 degrees Celsius, and the presence of meningeal signs. In the case of tick-borne encephalitis, there is no specific causal treatment available; therefore, symptomatic management remains the primary approach [20,28]. Even after complete recovery, patients often report persistent headaches, difficulties with concentration and memory, and mood disturbances. The most frequent subjective sequelae were headaches, cognitive sequelae—memory impairment, psychiatric sequelae, etc.—depression, and neurological sequelae—like upper limb paresis [32].

Given that the treatment for viral neuroinfections primarily focuses on managing symptoms, it is crucial to prioritize both the diagnosis and pharmacological management of neurodegenerative processes associated with these conditions. Addressing neurodegeneration adequately may significantly contribute to preventing the emergence of adverse neurological outcomes following viral neuroinfections. Many studies have observed that calcium ion levels correlate with the stage of degeneration or the occurrence of psychiatric disorders. In inflammatory and neurodegenerative processes, the concentration of calcium in glial cells is regulated by inflammatory mediators. An increase in calcium levels within astrocytes and other glial cells is associated with chronic inflammation, which contributes to neurodegeneration [33]. This finding raises hopes for identifying another potential biomarkers that could assist in the early diagnosis of neurodegenerative changes at a stage where they may not yet manifest fully.

Further research is needed to find answers to key questions and to assess the role of calcium homeostasis in neuroinflammation processes due to tick-borne encephalitis infections.

Calcium plays a crucial role in numerous biological processes, including the functioning of the nervous system. The concentration of calcium in the cerebrospinal fluid (CSF) is tightly regulated and typically maintained at a constant level. This calcium homeostasis is essential for the proper functioning of neurons, as calcium is involved in signal transmission, neurotransmitter release, and various other cellular processes.

Discrepancies in the calcium concentration within the CSF may indicate potential disturbances in calcium homeostasis, which, in the context of the central nervous system, can signify the onset of neurodegenerative processes [34]. In the context of neuroinflammation, disruptions in calcium signaling pathways can trigger the activation of microglia and astrocytes, subsequently leading to the release of pro-inflammatory cytokines and further neuronal injury. Calmodulin, a protein that binds calcium, plays a pivotal role in modulating these inflammatory and neurodegenerative processes [35]. Monitoring calcium levels in the CSF, therefore, can serve as an important diagnostic tool for the early detection and tracking of neurodegenerative diseases. Early diagnosis and intervention may potentially delay disease progression and improve the quality of life for patients [36].

Cognitive and attention impairments can arise from both hypocalcemia and hypercalcemia. Severe disturbances of consciousness are most commonly observed when serum calcium levels exceed 14 mg/dL [37,38]. Nevertheless, most hypercalcemic patients experience only mild to moderate elevations in blood calcium, with neurological symptoms generally presenting as headaches, lethargy, proximal muscle weakness in the lower limbs, confusion, and intense muscle pain [36].

Patients with calcium homeostasis disorders frequently present with neuropsychiatric symptoms, including delusions, delirium, agitation, and hallucinations. Other clinical manifestations include involuntary movements like chorea or choreoathetosis seen in hypocalcemia, and bradykinesia indicative of parkinsonism in hypercalcemia. A decrease in calcium levels can also result in seizures. EEG changes observed in calcium homeostasis disorders may show progressive generalized or focal slowing of brain activity and other signs of metabolic encephalopathy, such as triphasic waves, which gradually disappear once blood calcium levels are normalized [36]. Changes in serum calcium levels often correlate with systemic conditions; calcium levels in cerebrospinal fluid (CSF) are regulated independently. The blood–brain barrier plays a critical role in maintaining this separation, ensuring that fluctuations in serum calcium do not directly translate to changes in CSF calcium. This unique regulation underscores the importance of investigating CSF calcium as a biomarker, as it may provide distinct insights into neurological processes, including those associated with TBE [39,40,41].

In our study, we obtained intriguing results. The concentration of calcium in the cerebrospinal fluid was significantly lower than in the control group, with the lowest levels observed in the mild course group. This may be characteristic of the disease entity, such as TBE, but further studies on a larger group of individuals are necessary to confirm this.

We demonstrated that the level of calcium is dependent on the clinical presentation. This finding suggests that calcium levels could become a predictor of disease progression in its early stages, which is crucial for selecting the appropriate therapeutic regimen. Consequently, we could potentially forecast the course of the disease at the time of admission for each patient. Moreover, dysregulation of calcium homeostasis may be responsible for the development of various complications, including those at the interface of psychiatry and neurology [42,43].

The control group, consisting of patients admitted to the Department of Infectious Diseases and Neuroinfections with meningitis excluded, had calcium levels in their CSF ranging from 0.86 to 1.06 mm/L. These levels were predominantly below the normal range specified in the introduction (1.047–1.247 mm/L). It is worth noting that the slightly reduced calcium levels in this group may be attributable to comorbid conditions or the specific health issues that prompted their admission to the clinic. The most common diagnoses leading to their referral were ’headache’ and ‘muscle and joint pain’, which could reflect a variety of underlying causes potentially influencing calcium homeostasis.

Calcium concentration in the CSF remains relatively stable, making any deviations from the reported ranges potentially significant for diagnostic purposes. We anticipated elevated calcium levels; however, the results are, at the very least, thought-provoking. At this stage, it is difficult to establish a clear connection between calcium levels and neurodegenerative processes. Further follow-up studies are necessary to determine whether calcium levels change over time. It is also important to compare the obtained results with potential psychiatric disorders presented by the patients, such as depressive states, anxiety disorders, or cognitive impairments.

Advantages of the study include the following: investigation into an endemic disease; the exploration of potential new markers for predicting disease progression; the low cost of the study; the ease of result replication; enhanced understanding of neurodegenerative processes associated with TBE; and the novelty of the study: this study is the first to evaluate calcium levels in the cerebrospinal fluid of patients with TBE, which may open new avenues for research into the pathogenesis and diagnosis of this disease.

Disadvantages of the study include the following: the relatively small sample size, which is attributable to the low prevalence of the disease, and the lack of precisely established reference ranges for calcium concentration in cerebrospinal fluid in healthy adults, although this can be addressed through further research. What is more, as the study was based on previously gathered and stored samples (retrospective analysis) we assessed only total calcium concentration, while ionized calcium concentration could provide more adequate results.

Additional considerations: the small sample size, while a limitation, provides an opportunity for future studies to validate and expand upon the findings. The establishment of reference ranges for calcium levels in cerebrospinal fluid could enhance the robustness of future research and clinical applications.

## 6. Conclusions

Calcium levels: The study reveals a statistically significant difference in calcium levels between patients with mild and severe tick-borne encephalitis (TBE), with lower calcium levels observed in those with a mild illness course. Monitoring calcium levels could provide valuable information for assessing disease severity and tailoring treatment strategies. We recommend integrating calcium level assessments into routine clinical practice, especially for patients presenting with neurological symptoms or suspected TBE. This could help in early detection, prognosis assessment, and personalized therapeutic interventions.

ROC Analysis: The area under the curve (AUC) of 0.802 indicates that calcium concentration serves as a strong diagnostic marker for distinguishing between varying severities of TBE. The high sensitivity and specificity of calcium levels at certain thresholds further reinforce its potential diagnostic value. The proposed cut-off at 3.09 mg/dL is not obligatory but merely states the point at which overall sensitivity and specificity seems optimal; however, it can be changed when either better specificity or sensitivity is needed.

Future Directions: Additional research is needed to identify the most effective biomarker for improving clinical management of TBE. Such advancements could lead to shortened hospital stays through the early implementation of targeted therapies and a reduction in potential complications. To validate our findings, we plan to conduct studies with a larger sample size to enhance the statistical power and reliability of the results. Multi-center trials would indeed be an excellent approach to ensure a more diverse and representative cohort.

Call to Action: To further our understanding of TBE and its relationship with calcium homeostasis, researchers, clinicians, and public health officials must collaborate to address the gaps identified in this study. Researchers should pursue larger-scale studies to explore the impact of calcium dysregulation on TBE and neurodegenerative outcomes. Clinicians are encouraged to incorporate calcium level assessments into diagnostic and monitoring protocols to enhance disease management and treatment personalization. Public health officials should advocate for continued research funding and support initiatives aimed at clarifying the role of calcium imbalances in neuroinflammatory diseases.

## Figures and Tables

**Table 1:** Characteristics of group.

	TBE (*n* = 20)	Other Etiology (*n* = 11)	Control Group (*n* = 11)	Overall	p TBE vs. Other	p TBE vs. CG	p Other vs. CG
Mean age [years]	47.6	48.91	39.27	45.76	0.17	0.14	0.9
Total number of woman (%)	9 (45%)	5 (45.45%)	6 (54.55%)	20 (47.62%)	0.98	0.61	0.67
Total number of man (%)	11 (55%)	6 (54.55%)	5 (45.45%)	22 (52.38%)			

p TBE vs. Other: comparison of analyzed parameters between the TBE group (group 1) and the other etiology group (group 2). p TBE vs. CG: comparison of analyzed parameters between TBE group (group 1) and control group (group 3). p Other vs. CG: comparison of analyzed parameters between other etiology group (group 2) and control group (group 3).

**Table 2:** Calcium levels in CSF.

	TBE (*n* = 20)		Other Etiology (*n* = 11)		Control Group (*n* = 11)		p TBE vs. Other	p TBE vs. CG	p Other vs. CG
	[mmol/L]	[mg/dL]	[mmol/L]	[mg/dL]	[mmol/L]	[mg/dL]			
Mean calcium level	0.85	3.42	0.92	3.69	0.98	3.91	ns	0.03	ns
Median calcium level	0.89	3.57	0.92	3.69	0.99	3.97			
Standard deviation (SD)	0.16	0.64	0.09	0.36	0.06	0.24			
Min	0.55	2.21	0.76	3.05	0.86	3.45			
Max	1.04	4.17	1.08	4.33	1.06	4.25			

p TBE vs. Other: comparison of analyzed parameters between TBE group (group 1) and other etiology group (group 2). p TBE vs. CG: comparison of analyzed parameters between TBE group (group 1) and control group (group 3). p Other vs. CG: comparison of analyzed parameters between other etiology group (group 2) and control group (group 3). ns: non-significant.

**Table 3:** Comparison of calcium levels in CSF between groups with mild and severe courses of TBE.

	Mild (*n* = 7)		Severe (*n* = 13)		p Mild vs. Severe
	[mmol/L]	[mg/dL]	[mmol/L]	[mg/dL]	
Mean calcium level	0.75	3.00	0.91	3.65	0.03
Median calcium level	0.71	2.85	0.96	3.85	
standard deviation (SD)	0.16	0.65	0.13	0.53	
min	0.55	2.21	0.57	2.29	
max	0.97	3.89	1.04	4.17	

p Mild vs. Severe: comparison of analyzed parameters between TBE group (group 1) and other etiology group (group 2).

## Data Availability

The datasets used and analyzed during the current study are available from the corresponding author on reasonable request.
